# Sources of exposure identified through structured interviews of healthcare workers who test positive for severe acute respiratory coronavirus virus 2 (SARS-CoV-2): A prospective analysis at two teaching hospitals

**DOI:** 10.1017/ash.2021.243

**Published:** 2021-12-15

**Authors:** Chanu Rhee, Meghan A. Baker, Robert Tucker, Diane Griesbach, Dinah McDonald, Sarah A. Williams, Karen Fiumara, Andrew Resnick, Michael Klompas, for the CDC Prevention Epicenters Program

**Affiliations:** 1 Department of Population Medicine, Harvard Medical School/Harvard Pilgrim Health Care Institute, Boston, Massachusetts; 2 Division of Infectious Diseases, Department of Medicine, Brigham and Women’s Hospital, Boston, Massachusetts; 3 Infection Control Department, Brigham and Women’s Hospital, Boston, Massachusetts; 4 Occupational Health Services, Brigham and Women’s Hospital, Boston, Massachusetts; 5 Occupational Health Services, Brigham and Women’s Faulkner Hospital, Boston, Massachusetts; 6 Department of Quality and Safety, Brigham and Women’s Hospital, Boston, Massachusetts

## Abstract

We interviewed 1,208 healthcare workers with positive SARS-CoV-2 tests between October 2020 and June 2021 to determine likely exposure sources. Overall, 689 (57.0%) had community exposures (479 from household members), 76 (6.3%) had hospital exposures (64 from other employees including 49 despite masking), 11 (0.9%) had community and hospital exposures, and 432 (35.8%) had no identifiable source of exposure.

Healthcare workers (HCWs) are presumed to be at high risk for severe acute respiratory coronavirus virus 2 (SARS-CoV-2) infection.^1–5^ Studies based on self-reports and serologic testing, however, differ on whether HCW infection rates are higher or similar to rates in surrounding communities and how many infections are acquired inside versus outside the hospital. Importantly, many HCW studies were conducted early in the pandemic when personal protective equipment (PPE) was scarce, testing and infection control strategies were still evolving, and vaccines were not yet deployed. We report on a prospective analysis of HCW infections based on structured interviews at two hospitals in Boston, Massachusetts, beginning in October 2020 when coronavirus disease 2019 (COVID-19) infection control policies had matured and PPE supplies had stabilized.

## Methods

### Setting, employee testing, contact tracing, and exposure evaluation process

We included all employees at Brigham and Women’s Hospital (803-bed academic hospital) and Brigham and Women’s Faulkner Hospital (162-bed community teaching hospital) who tested positive for SARS-CoV-2 between October 1, 2020, and June 1, 2021. These 2 hospitals collectively employ >23,000 people. Employees were required to undergo PCR testing for any symptoms potentially consistent with COVID-19, if they had a known exposure in the community, or if they interacted with a SARS-CoV-2–positive employee with ≥1 party unmasked for ≥15 minutes while that person was potentially infectious (2 days prior to symptom onset or date of positive test if asymptomatic). Employees who cared for SARS-CoV-2–positive patients before they were placed on COVID-19 precautions were identified through the Epic software (Epic, Verona WI) electronic health record trace function and were notified by e-mail. They were required to test if they were within 2 m of the patient for ≥15 minutes unless both parties wore face masks or the employee wore both a mask (or respirator) and eye protection (face shield or goggles). Asymptomatic employees were also permitted to test on or off site at no cost for any other reason.

All positive employees were immediately interviewed by the occupational health department to ask about presence and timing of symptoms, interactions with patients or staff while potentially infectious, and potential exposure sources in the preceding 14 days. Employee contacts were notified by e-mail or phone if the SARS-CoV-2–positive staff member reported that exposure criteria were met (≥1 party unmasked for ≥15 minutes during the infectious period). Each SARS-CoV-2–positive case was then discussed on daily multidisciplinary calls involving Infection Control, Quality and Safety, and Occupational Health staff. The likely exposure source was adjudicated as community, hospital, mixed, or unknown using case definitions that considered known COVID-19 contacts, whether employees worked on site in the preceding 14 days, whether full PPE was used during care for COVID-19 patients (ie, respirator, eye protection, gown, gloves), and whether masks were worn (Table 1).

**Table 1.** Criteria for Classifying Source of Healthcare Worker Infections

Classification	Criteria
**Community source**	
Definite community exposure	• Close contact (within 2 m) with someone outside of the hospital for ≥15 minutes who is confirmed to be SARS-CoV-2 positive within the preceding 14 d, absent a similar exposure within the hospital, OR • Did not work on-site within the 14 d preceding symptom onset
Likely community exposure	• Household contact and employee develop symptoms at the same time and no known work-related exposure, OR • Household contact displaying COVID-19 symptoms but not tested, and no known work-related exposure, OR • Traveled out of state within 14 d of symptom onset and no known contact with a SARS-CoV-2–positive patient or staff member at work
**Hospital source**	
Patient exposure with inappropriate PPE	• Did not meet criteria for community exposure, AND • Close contact (within 2 m) for ≥15 minutes within the prior 14 d with a SARS-CoV-2–positive patient from 2 d prior to symptom onset (or positive test if asymptomatic) to the time patient was placed on precautions, in which the employee was not wearing full PPE (respirator, eye protection [face-shield or goggles], gown, and gloves)
Employee exposure	• Did not meet criteria for community exposure, AND 1 of the following: • *Unmasked:* Close contact (within 2 m) within the prior 14 d with a SARS-CoV-2–positive employee anytime from 2 d prior to symptom onset to the time the SARS-CoV-2–positive employee left work, in which either employee was not wearing a mask for ≥15 minutes • *Masked*: As above, but no reported lapses in mask use
**Mixed community/hospital source**	
Mixed exposures	• Cared for SARS-CoV-2–positive patient(s) without appropriate PPE OR had an employee exposure (with or without lapses in mask use) within the prior 14 d, AND • Close contact with someone outside the hospital who tested positive with onset of symptoms on the same day or within 24 h prior to the employee or displayed COVID-19 symptoms but not tested
**Unknown source**	
Patient exposure with appropriate PPE	• Did not meet criteria for community or hospital exposure, AND • Employee had close contact (within 2 m) for ≥15 minutes within the prior 14 d with a SARS-CoV-2–positive patient while wearing full PPE (respirator, eye protection (face-shield or goggles), gown, and gloves)
No known exposures	• No contacts with known or suspected COVID-19 individuals in or out of the hospital within the prior 14 d, and not meeting any of the definitions above

Employees whose only contacts were with COVID-19 patients while wearing full PPE were classified as having an “unknown” exposure source given the evidence that transmission to HCWs wearing full PPE is exceedingly rare.^6^ Employees whose only known COVID-19 contacts were other employees were classified as “hospital” exposures even if both reported wearing surgical masks given our observations that minor lapses in mask use (ie, uncovered noses, sipping beverages) during HCW-HCW interactions are not uncommon, and data that transmission can occur despite surgical masks.^7–9^

### Patient testing and infection control policies

All patients underwent SARS-CoV-2 PCR testing on admission; PCR-negative patients were retested 72 hours later to identify virus potentially incubating on arrival and again every 3 days through day 14 if they required aerosol-generating procedures. Serial testing was also done for all patients on a unit if a hospital-onset case or potential cluster was detected. Liberal retesting was encouraged for any new symptoms concerning for COVID-19. Precautions for patients with suspected or confirmed COVID-19 included respirators, eye protection, gowns, and gloves. Standard precautions for non–COVID-19 patients included surgical masks and eye protection. A universal mask policy for all employees was in effect.

### Analysis

We analyzed the distribution of likely exposure sources among SARS-CoV-2–positive employees overall and stratified by vaccination status using the Cochran-Mantel-Haenszel statistic. The hospitals began administering the BNT162b2(Pfizer/BioNTech) and mRNA-1273 (Moderna) vaccines in mid-December; 85% of all staff were vaccinated by June 1. We considered employees vaccinated if they received ≥1 dose of any vaccine ≥14 days before infection given the evidence of at least partial immunity by this point.

All analyses were conducted using Microsoft Excel 365 software (Microsoft, Redmond, WA) and SAS version 9.4 software (SAS Institute, Cary, NC). The study was approved by the Mass General Brigham Institutional Review Board.

## Results

**Fig. 1.**
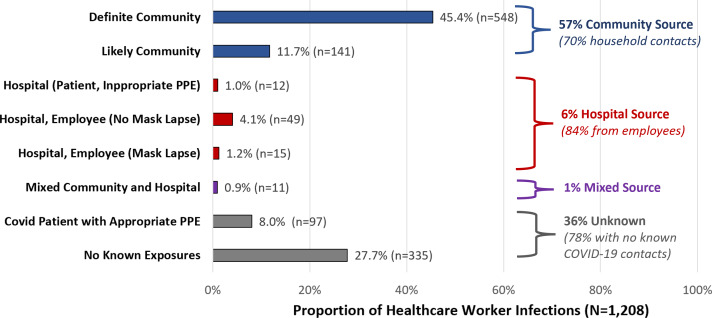
Likely sources of exposure for healthcare workers with positive SARS-CoV-2 tests based on structured occupational health interviews.

During the study period, 12,228 employees underwent 53,422 tests and 1,208 tested positive for SARS-CoV-2 (2.3% positivity rate), including 121 providers (physicians, physician assistants, nurse practitioners), 324 nurses, 166 patient care or medical assistants, 219 other clinical staff, and 378 nonclinical staff. Also, 1,004 (83.1%) employees were symptomatic when tested. Overall, 689 (57.0%) employees had community exposures (548 definite and 141 likely), including 479 with household contacts. In addition, 76 (6.3%) had hospital exposures: 12 from COVID-19 patients before they were diagnosed and placed on COVID-19 precautions, and 64 from other employees including 49 who reported both parties wore masks. Furthermore, 11 (0.9%) had both community and hospital exposures. Importantly, 432 (35.8%) had an unknown source: 97 who cared for COVID patients wearing full PPE, and 335 with no known COVID-19 contacts (Fig. 1).

Of 1,208 SARS-CoV-2–positive employees, 90 (7.5%) tested positive ≥14 days after their first vaccine dose. Exposures sources for these vaccinated employees were respectively similar to unvaccinated SARS-CoV-2–positive employees: community (63.3% vs 56.5%), hospital (0% vs 6.8%), mixed (1.1% vs 0.9%), and unknown (35.6% vs 35.8%) (*P* = .65).

## Discussion

Overall, 57% of HCWs who tested positive for SARS-CoV-2 between October 2020 and June 2021 had a known community exposure, mostly household contacts. An additional 6% had hospital exposures, mostly from other HCWs (23% with unmasked interactions), and 1% had mixed community and hospital exposures. Strikingly, we were unable to identify a clear exposure source in 36% of cases despite detailed interviews. Most had no known COVID-19 contacts; one-quarter cared for COVID-19 patients but with appropriate PPE. Sources of infection were similar for unvaccinated and vaccinated HCWs.

Our findings complement seroprevalence studies demonstrating that most HCW infections are community acquired.^1,4,5^ Our analysis does suggest, however, that ∼10% of HCW infections with a known source are potentially attributable to the workplace, particularly from peers and patients with occult or not-yet-diagnosed infections.

The high rate of infections without an identifiable source may reflect the large number of asymptomatic SARS-CoV-2 infections and the fact that many cases in the community go untested and undiagnosed. We presume that many of these HCW infections were acquired in the community given that SARS-CoV-2–positive contacts in the hospital, particularly patients, are more likely to be recognized due to extensive testing and contact tracing. However, it is also possible that we underestimated workplace transmission because hospital policy did not require routine surveillance testing for asymptomatic HCWs and because occupational health staff did not routinely alert HCWs about SARS-CoV-2–positive peers if the peer reported both parties wore masks, despite the known possibility of transmission despite surgical masks.^7–9^ Furthermore, our exposure definition required ≥15 minutes of close contact with a SARS-CoV-2–positive individual, but transmissions can occur over shorter intervals.^7,9^ These policies and exposure definitions may have made it more difficult to identify workplace exposures.

Our findings are largely concordant with a University of Wisconsin study in which whole-genome sequencing was conducted for 95 HCWs and 137 possible patient contacts. These researchers demonstrated that most HCW infections were community-acquired, but one-quarter were work related, mostly from peers or patients in the context of unsuspected clusters rather than from known SARS-CoV-2–positive patients.^6^ This analysis and ours suggest that although workplace transmission likely accounts for a minority of HCW infections, there is still room to further enhance hospital infection control policies. Such interventions could include reminding HCWs of the risk of infection from asymptomatic colleagues and patients, minimizing high-risk unmasked interactions in breakrooms and workrooms, ensuring tight fit of surgical masks, and broader use of N95 respirators when community incidence rates are high.^7,10^ Furthermore, the large fraction of HCW infections without an identifiable source in our analysis underscores a potential benefit of conducting broader and more routine genomic sequencing of patient and HCW isolates. This could help to identify occult transmission pathways and areas of vulnerability, or conversely, provide reassurance about the effectiveness of current hospital infection control policies.

Limitations of our study include the focus on 2 well-resourced hospitals, potentially limiting generalizability, and our inability to use whole-genome sequencing to confirm transmission sources. The likelihood of community SARS-CoV-2 acquisition, particularly from unknown sources, may also vary depending on local public health measures such as mask mandates. Interviews are also subject to recall bias and some staff may have been reluctant to acknowledge lapses in mask use or high-risk behaviors. Lastly, our analysis was conducted before the highly transmissible δ (delta) variant began to predominate in the United States; additional research is needed to understand the impact of the δ variant on the adequacy of current infection control measures, particularly as vaccines may have reduced efficacy against this variant. Nonetheless, our findings provide important context to inform ongoing discussions and policies on how best to protect HCWs from SARS-CoV-2.
